# The effect of seminal fluid gene expression on paternity

**DOI:** 10.1093/evlett/qrad033

**Published:** 2023-08-07

**Authors:** Leigh W Simmons, Maxine Lovegrove

**Affiliations:** Centre for Evolutionary Biology, School of Biological Sciences (M092), The University of Western Australia, Crawley 6009, Australia; Centre for Evolutionary Biology, School of Biological Sciences (M092), The University of Western Australia, Crawley 6009, Australia

**Keywords:** fertilization success, RNA interference, seminal fluid proteins, sperm competition, *Teleogryllus oceanicus*

## Abstract

When females mate with more than one male, competition between rival ejaculates is expected to favor adaptations that promote fertilization success. There is now compelling evidence that sperm competition selects for increased production and allocation of sperm. However, sperm comes packaged in ejaculates that also contain protein-rich seminal fluids. Predicting how males should allocate individual seminal fluid proteins in response to sperm competition is hampered by our limited knowledge of their precise function. We use gene expression studies and interference RNA to ask how seminal fluid proteins in the ejaculate of a cricket, *Teleogryllus oceanicus*, affect a male’s paternity share when in competition for fertilizations. We find that the relative expression of one seminal fluid gene, *gagein*, positively affects the paternity share of competing males and that knockdown of this and two other seminal fluid protein genes renders males mating in the offensive role of sperm competition incapable of fathering living offspring. Despite having a negative effect on offspring viability these seminal fluid genes have been found to be up regulated in response to rival males, consistent with a role in promoting competitive fertilization success. Our data contribute to a growing body of evidence that, like sperm, seminal fluid gene expression is subject to post-mating sexual selection via sperm competition.

## Introduction

When females mate with more than one male, the sperm of rival males will often compete to fertilize a limited supply of ova, and sperm competition is expected to favor adaptations in the male ejaculate that promote competitive fertilization success (Parker, 1970; Simmons, 2001). Sperm competition game theory predicts that across species and populations, the selection from sperm competition should favor increased expenditure on sperm production and allocation (Parker & Pizzari, 2010). Sperm competition games have garnered considerable empirical support. Among species, the males of species with stronger selection from sperm competition have larger testes, produce more sperm, and sperm of greater length (for a recent meta-analysis of the literature, see Lüpold et al., 2020). Within species, males that perceive an increased risk of sperm competition (the probability that a female will mate with a rival male) will increase their immediate allocation of sperm to their current mate (Gage, 1991; Gage & Baker, 1991; Kelly & Jennions, 2011). While the evidence is clear that sperm competition promotes the evolution of sperm production and allocation, seminal fluid, the non-sperm component of the ejaculate, can play an equally important role in promoting a male’s competitive fertilization success and is also expected to evolve in response to sperm competition (Perry et al., 2013; Ramm, 2020; Simmons & Fitzpatrick, 2012).

There is now good evidence from a variety of taxa that seminal fluid can promote the motility or viability of sperm (Cornwallis & O’Conner, 2009; den Boer et al., 2008; Holman, 2009; Liberti et al., 2018; Poli et al., 2018) and their competitive fertilization success (Bartlett et al., 2017), that seminal fluid protein (sfp) genes are subject to positive selection (Clark et al., 2006; Swanson & Vacquier, 2002), and that sfp gene expression (Fedorka et al., 2011; Nakadera et al., 2019; Simmons et al., 2019) and the protein composition of the seminal fluid (Goenaga et al., 2015; Ramm et al., 2015; Wigby et al., 2009) can vary in response to sperm competition risk. Attempts have been made to develop theoretical models with which to predict how males should invest in their seminal fluid composition in response to sperm competition. However, unlike sperm competition games, general predictions are hampered by the varied roles that individual seminal fluid proteins can play in reproduction (Alonzo & Pizzari, 2010; Cameron et al., 2007; Dhole & Servedio, 2014). Many sfps have been identified that promote female fecundity (Gillott, 2003; Simmons, 2001). Seminal fluid games predict that increased risk of sperm competition could favor reduced investment in fecundity stimulating sfps if a male loses paternity share to sperm competition rivals (Cameron et al., 2007), but complex coevolutionary interactions between females and males can arise when sfps also affect female remating and rival males share in the benefits of fecundity stimulation (Alonzo & Pizzari, 2010). When seminal fluid proteins enhance sperm competitive ability, seminal fluid games predict that males should increase their allocation to sfps in response to sperm competition (Cameron et al., 2007; Dhole & Servedio, 2014). However, exactly how males should invest in their sfps depends on the role these proteins play in the avoidance of sperm competition, defense against rival males, or offense against a female’s previous mating partners (Dhole & Servedio, 2014). Testing these predictions is thereby hampered by our limited understanding of the functional role of individual sfps within the ejaculate.

Seminal fluid can comprise several hundreds of different proteins whose individual functions in reproduction remain largely unknown (Baer et al., 2009; Druart et al., 2013; Kelly et al., 2006; Pilch & Mann, 2006). Our best understanding comes from studies of *Drosophila melanogaster* in which sfps that affect fecundity, female receptivity to remating, and competitive fertilization success in offensive and defensive roles have been identified, either via association studies or functional genomics (Wigby et al., 2020; Wolfner, 2002). However, while almost 300 proteins have been identified in the *Drosophila* proteome, only ~5% of these 300 proteins have so far been functionally characterized and found to affect competitive fertilization success using gene knockdown procedures (Patlar & Civetta, 2022; Wigby et al., 2020). These seminal fluid proteins appear to be involved in the storage and release of sperm received by the female from her first mating partner, as they affect the competitive fertilization success of the first (defensive) but not the second (offensive) male to mate (Patlar & Civetta, 2022). Far less is known outside of *Drosophila*. An association study of the seed beetle *Callosobruchus maculatus* implicated sfps in promoting competitive fertilization success because the divergence in the seminal fluid proteome was correlated with divergence in competitive fertilization success (Goenaga et al., 2015). Recent work has also identified two proteins in the seminal fluid of the hermaphroditic flatworm *Macrostoma lignano*, Suckless-1 and Suckless-2, that inhibit a post-mating behavior in which the recipient worm sucks donor sperm from its gonopore, and so prevents sperm transport and storage; knockdown of *suckless-1* and *suckless-2* results in reduced donor paternity success because knockdown individuals are unable to inhibit recipient sucking behavior (Patlar & Ramm, 2020; Patlar et al., 2020).

Functional genomic studies such as RNAi knockdown are useful in identifying the causal effects of sfps on competitive fertilization success, where association studies are unable to identify cause and effect (Wigby et al., 2020). However, genomic manipulations cannot reveal the strength or direction of post-mating sexual selection acting on sfps, which require an estimation of the effects of natural variation in sfp gene expression on the competitive fertilization success phenotype. Here, we employ a parallel approach to examine the effect of sfp gene expression on paternity outcome in a field cricket, *Teleogryllus oceanicus*. The seminal fluid of *T. oceanicus* affects the viability of sperm contained within the ejaculate (Simmons & Beveridge, 2011). Some 27 proteins have been identified from the seminal fluid (Simmons et al., 2013, 2014a), among which seven have been implicated to play a role in sperm competition because their expression levels are adjusted plastically in response to the presence of rival males, with increased sfp gene expression being associated with increased sperm viability (Simmons & Lovegrove, 2017). Functional genomic studies using RNA interference have shown that at least one protein, ToSfp011, hereafter denoted as Gagein,^1^ appears to have pleiotropic effects on sperm viability (Simmons & Lovegrove, 2017), post-mating female sexual receptivity (Moschilla et al., 2020) and the prenatal viability of developing embryos (Simmons & Lovegrove, 2020). Knockdown of two additional sfp genes, *ToSfp001* and *ToSfp017*, also reduce sperm viability and are upregulated in response to sperm competition risk (Simmons & Lovegrove, 2017). While sperm viability does contribute to the competitive fertilization success of *T. oceanicus* males (García-González & Simmons, 2005), no direct link between paternity and sfp gene expression has yet been established. In this study, we determine the effect of natural variation in sfp gene expression of rival males on their paternity share when in post-mating competition. We also use interference RNA to knockdown sfp genes individually, in order to determine the effects of these sfps on paternity when males mate in the offensive role of the second male.

## Methods

Male crickets used in these experiments were derived from a large (>1000 individuals) outbred stock originating from fruit plantations in and around Carnarvon, Western Australia. The population is seeded annually with ~50 newly collected adult crickets and is maintained in a constant temperature room held at 26 °C with a 12 hr:12 hr light:dark cycle. The stock culture was checked daily for penultimate instar nymphs, which were housed individually in small plastic boxes (7 × 7 × 5 cm) and provided with cat chow and water ad libitum. Nymphs were checked daily until adult emergence, and adult crickets were used in experiments when they were sexually mature, 7–14 days after the adult molt. Female *T. oceanicus* can have considerable effects on the fertilization success of competing males via differential storage and utilization of sperm (Simmons & Beveridge, 2010; Simmons et al., 2014b; Tuni et al., 2013). To minimize female effects in these experiments, females were drawn from generation 20 of a single isofemale line (described in Simmons & Lovegrove, 2019). The line was checked daily for penultimate instar females which were collected and raised individually as described for males and used in experiments 7–14 days after adult eclosion.

### Sperm competition trials

Sperm competition trials were carried out over a period of 2 days. At the start of the dark cycle on Day 1, a single male was removed from his individual container and placed into the container of a previously unmated female, and the pair observed until the male had transferred a spermatophore. After a successful mating, crickets were monitored closely to ensure that the spermatophore remained attached for a period of 40 min, the time necessary and sufficient for complete ejaculate transfer (Simmons et al., 2003). Males were then removed and frozen. At the start of the dark cycle on Day 2, each female was provided with a second male, and mating was observed as on Day 1. After the male was removed and frozen, females were provided with a dish containing damp cotton wool in which to oviposit and left for 7 days. Females were then frozen and the egg pads incubated for 14 days after which time emerging nymphs were collected and frozen for subsequent paternity analysis. We conducted 32 sperm competition trials in which the first and second males were unmanipulated and had a target of 15 sperm competition trials involving triads in which one of seven target seminal fluid genes had been knocked down in the second male using interference RNA.

### Interference RNA

We examined the effect of seven seminal fluid protein genes on paternity. These genes were chosen because they were found elsewhere to be upregulated in response to social cues of sperm competition risk, and so were predicted to play a role in competitive fertilization success (Simmons & Lovegrove, 2017). The double-stranded RNA required for gene knockdown was prepared using a Megascript RNAi kit (Life Technologies). We used the same PCR primers designed for a previous RNAi experiment with the T7 promotor sequence added to the 5ʹ end of each primer (see Supplementary Table S1; Simmons & Lovegrove, 2017). Template DNA was prepared in four identical polymerase chain reactions (PCRs), each 50 µl reaction containing 1× PCR buffer (10 mM Tris-HCl pH 8.3, 50 mM KCl), 3 mM MgCl_2_, 200 µM of each dNTP, 250 nM of forward primer, 250 nM of reverse primer, 10% DMSO, 2.5 units of Platinum Taq polymerase and 100 ng cDNA obtained using RNA extracted from a cricket accessory gland (all reagents were from Thermofisher Scientific). PCR amplification was performed with a two-step PCR reaction with cycling conditions as follows: 94 °C for 3 min, then 35 cycles of 94 °C for 30 s and 68 °C for 60 s, then finally, 72 °C for 5 min. The four PCR reactions were pooled and purified using two columns of the Favorgen PCR clean-up kit (Fisher Biotech) with elution into 2 × 40 µl. These were pooled, and the DNA concentration was determined using a Nanodrop 1000. The DNA was of a high enough concentration to be used to generate dsRNA. The Megascript RNAi kit (Thermofisher Scientific) was used to generate dsRNA via T7 RNA transcription using the manufacturer’s protocol. Each transcription reaction contained 1 µg template DNA, and the transcription time was 4 hr. The excess DNA and ssRNA were removed, and the dsRNA purified followed the manufacturer’s instructions with the final elution in 100 µl. A small amount was diluted 1 in 10 and run on a 3% agarose gel to check the size and integrity of the dsRNA. The concentration was also checked using the Nanodrop 1000. The volume was adjusted to a final concentration of 1 µg/µl in the elution buffer.

On the first day of the penultimate instar, when male nymphs were sampled from the stock culture and segregated into individual boxes, a sample of 15 was injected with 2 µg dsRNA for one of the seven genes under test, a total of 105 male nymphs. An equivalent number of male nymphs were injected with elution buffer only (from the Megascript kit). These males were to be used as sperm competition rivals for the RNAi males. Males were checked daily, and on adult emergence, each male was reinjected with another 2 µg dsRNA or elution buffer. Six days after adult emergence, each male was provided with a female from the stock culture to ensure they were capable of mating. After a successful test mating, the injected male was used in a sperm competition trial the following day, as described above. RNAi males were always mated second, and a male injected with elution buffer was mated first. Thus, we assess the role of these seminal fluid proteins in sperm competition offense. Although we aimed for 15 triads for each of the seven genes, deaths and mating failures resulted in variation in sample sizes: *ToSfp001* (12), *ToSfp005* (11), *gagein* (12), *ToSfp017* (13), *ToSfp022* (5), *ToSfp023* (10), and *ToSfp027* (11).

### Gene expression

Gene expression assays were conducted on males used in our sperm competition trials. For the trials involving unmanipulated males, both males were assessed for all seven genes. For the trials involving knockdown males, a sample of 10 males (only 5 available for *ToSfp022*) from each gene knockdown treatment and the corresponding sperm competition rival were assessed only for the knockdown gene. All gene expressions were assayed relative to the housekeeping gene, *actin* (Simmons & Lovegrove, 2017).

Males were thawed, and the accessory gland dissected and placed in RNA*later* (Thermofisher Scientific). After storage overnight at 4 °C, the accessory glands were kept at −20 °C until assay. RNA was extracted from the entire accessory gland using the PureLink RNA mini kit (Thermofisher Scientific) following the manufacturer’s instructions. The tissue was disrupted using a micropestle (Interpath) and a homogenizer (Thermofisher Scientific). DNA was removed using an on-column PureLink DNAse treatment (Thermofisher Scientific), and the RNA yield was quantified using a Nanodrop 1000. The accessory gland represents a significant component of somatic mass, some 16.4 ± 2.4 mg or 2.6% of total body mass, and provided a large yield of RNA (77.8 ± 22.1 μg). Two micrograms of each RNA sample was converted to cDNA in a 20 µl reaction volume using the high-capacity RNA-to-cDNA kit (Thermofisher Scientific), following the manufacturer’s instructions.

Custom TaqMan gene expression assays have previously been designed for the genes assayed in this experiment (Simmons & Lovegrove, 2017). The forward, reverse, and reporter sequences for these assays can be found in Supplementary Table S2. Standard curves for each gene were also generated to determine the amount of cDNA required, and for all of the genes assayed, this proved to be 10 ng cDNA. Gene expression assays were conducted in triplicate for each candidate gene and reference gene (*actin*) with negative controls (no cDNA) as follows: 1 µl cDNA (10 ng), 5 µl 2× TaqMan Gene expression master mix (Thermofisher Scientific) and 0.5 µl 20× TaqMan custom assay mix in a 10 µl reaction volume. The assays were run in compatible 96-well plates on a StepOne Plus Real-Time PCR system (Thermofisher Scientific) using the following cycling conditions: 50 °C for 2 min, 95 °C for 10 min and then 40 cycles of 95 °C for 15 s and 60 °C for 1 min. Results were analyzed by StepOne software v2.3 (Thermofisher Scientific) and exported into DataAssist v3.0 software (Thermofisher Scientific) for sample comparison using the comparative *C*_T_ method (Schmittgen & Livak, 2008). We calculated the difference in expression between the seminal fluid gene and the reference *actin* and took the logarithm to the base 2 (2^−ΔCT^) as our measure of gene expression.

### Paternity assignment

DNA was extracted from the hind femur muscle of both males and the female for each sperm competition triad, using a salt-based digestion buffer as described in Simmons et al. (2006). The DNA concentration was measured using a Nanodrop 1000 (Thermofisher), and the DNA was diluted 1:10 in water to an approximate concentration between 10 and 20 ng/µl. DNA was extracted from a sample of 24 whole offspring per female using the EDNA HISPEX tissue kit (Fisher Biotec) using the manufacturer’s instructions.

To assign paternity, DNA from both adults and offspring were initially screened using four microsatellite markers (loci *Totri9a*, *Totri55a*, *Totri57*, and *Totri78*) that were developed specifically for *T. oceanicus* (Beveridge & Simmons, 2005). The four loci were combined into one multiplexed polymerase chain reaction (PCR). Each 10 µl PCR reaction contained 1× Qiagen multiplex master mix (Qiagen), 2 µM of each forward and reverse primer, and 1 µl DNA (~10–20 ng). The forward primers were labeled with fluorescence (6-FAM, NED, VIC, and PET, respectively). PCR amplification was carried out in a Mastercycler Nexus gradient thermal cycler (Eppendorf) with cycling conditions as follows: 95 °C for 15 min, then 30 cycles of 94 °C for 30 s, 57 °C for 90 s and 72 °C for 60 s, and finally 72 °C for 30 min. The PCR products were analyzed on an ABI3730 Sequencer (Applied Biosystems), sized using Genescan-500 LIZ internal size standard, and genotyped using the software Genemarker v1.9 (Softgenetics LLC). For those samples where paternity could not be assigned, further genotyping was carried out using an additional multiplex PCR containing the following two loci, *Totri54* and *Totri59* (forward primers labeled with 6-FAM and VIC, respectively). PCR conditions and fragment analysis were described earlier. Parentage could not be assigned to 25 of 2592 offspring (0.9%). A large number (15) of these offspring came from one sperm competition triad, which was removed from all subsequent analyses.

### Statistical analyses

We compared the levels of expression for each sfp gene in unmanipulated first and second males using paired *t* tests. Paternity share is a relative measure of fitness obtained by two males competing within a single female. To examine the effect of sfp gene expression on the paternity shares for unmanipulated males, we therefore calculated relative gene expression as the difference in gene expression between competing males (second male to mate minus first male to mate). We entered values of relative gene expression for each of the seven genes into a generalized linear model in R (R_Core_Team, 2021), using a logit link function and the number of offspring sired by the second male to mate as the numerator and the total number of offspring genotyped for that female as the binomial denominator. The quasibinomial family was used to avoid overdispersion. We calculated McFaddon’s *R*^2^ to estimate how well the model fitted the data. The gene expression data for knockdown males were, as expected, zero skewed, and so we adopted nonparametric median tests to analyze these data in JMP15. Likewise, for sperm competition trials involving knockdown second males, there were a large number of cases in which the RNAi male obtained no fertilizations, and indeed the median value for second male paternity was zero for some genes. We, therefore, compare the paternity share for knockdown second males with that found in matings involving unmanipulated males using nonparametric median tests.

## Results

### Gene expression

**Figure 1.**
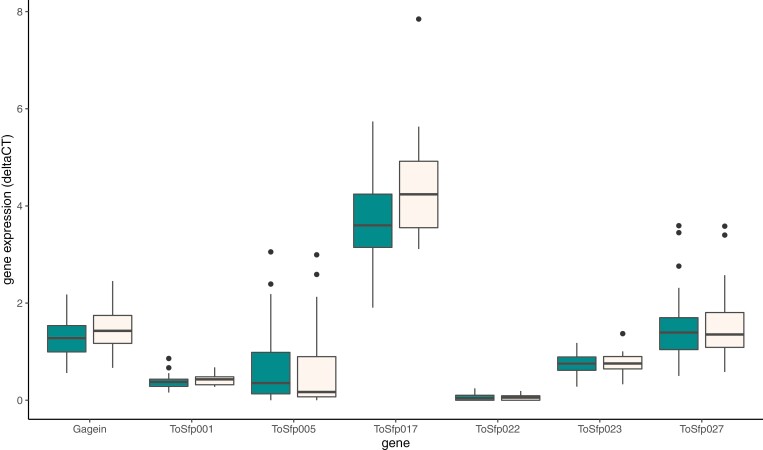
Boxplots of the expression (2^−ΔCT^) of seven seminal fluid protein genes in first (teal) and second (wheat) males in unmanipulated sperm competition trials. The median is indicated by the black horizontal bar, the box represents the upper and lower quartiles, whiskers are 1.5× interquartile range, and circles are outliers.

The expression levels for the seven seminal fluid protein genes are shown in Figure 1. We compared the expressions of the sfp genes of rival males by testing the mean relative expression against an expectation of zero (both first and second males have equal levels of sfp gene expression). The relative expressions of both *gagein* (*t* = 2.458, df = 31, *p* = .032) and *ToSfp017* (*t* = 2.593, df = 31, *p* = .009) were significantly greater than zero, indicating that males mating second in sperm competition trials had the greater sfp gene expression. The relative expressions of all other sfp genes did not differ significantly from zero (*t* = −0.849 to 1.679, df = 31, *p* = .103–.558).

### Paternity share in unmanipulated sperm competition trials

**Figure 2.**
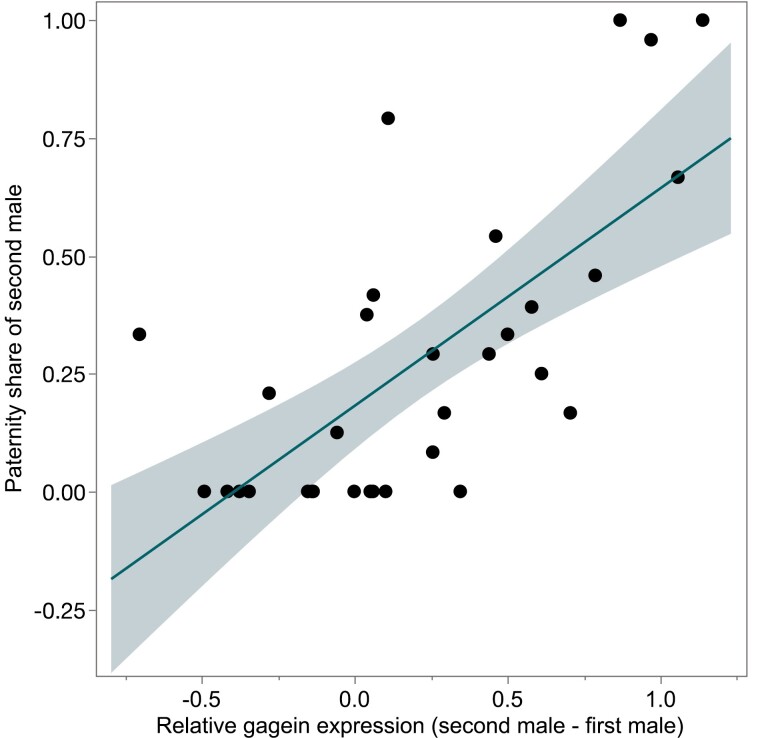
The proportion of offspring sired by the second of two males to mate in relation to the relative expressions of *gagein*. The regression line is presented with its 95% confidence intervals.

The median proportion of offspring sired by the second male to mate was 0.2 (interquartile range, 0–0.9). If the expression of sfp genes contributes to the competitive fertilization success of both males, we would expect the second male’s paternity share to increase as his sfp gene expression increased relative to the sfp gene expression of his rival. The relative expression of *gagein* had a significant effect on paternity outcome. The proportion of offspring sired by the second male to mate increased with increasing expression of *gagein* relative to his sperm competition rival (χ^2^ = 19.48, df = 1, *p* < .001, McFadden’s *R*^2^ = .44; Figure 2). No other seminal fluid protein genes affected paternity outcome and were removed from this final model (for full model statistics, see Supplementary Table S3).

### Interference RNA

**Figure 3.**
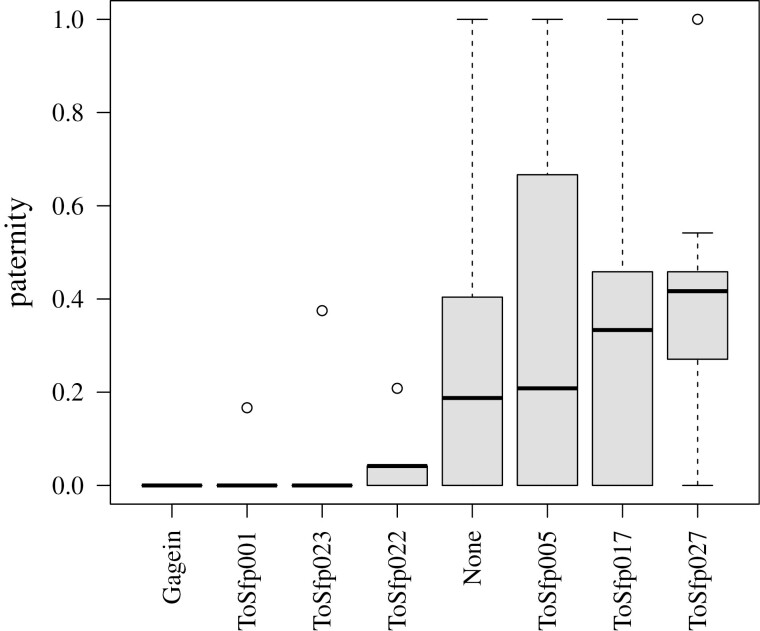
Boxplots of paternity (proportion of offspring sired) achieved by second males to mate when one of their seminal fluid protein genes had been knocked down using interference RNA. Shown also is the distribution of second male paternity for unmanipulated males that had none of their sfp genes knocked down. The median is indicated by the black horizontal bar, the box represents the upper and lower quartiles, whiskers are 1.5× interquartile range, and circles are outliers.

If sfps contribute to a male’s competitive fertilization success, knocking down the expression of sfp genes should have the effect of reducing a male’s paternity share. RNAi was successful in knocking down the expression of the seven seminal fluid protein genes (Table 1). Knockdown of *ToSfp001*, *gagein, ToSfp023*, and *ToSfp022* reduced a male’s share in the paternity of hatched offspring (Figure 3). Paternity shares for second males were significantly lower than expected from matings involving unmanipulated second males for *ToSfp001* (*p* = .002), *gagein* (*p* < .001), and *ToSfp023* (*p* = .036) knockdowns. The reduction in paternity share for *ToSfp022* knockdowns was not statistically significant (*p* = .162), but the test lacked statistical power as only five males survived the knockdown. Knockdowns for the remaining genes had similar paternity shares to unmanipulated second males (Figure 3).

**Table 1.** Seminal fluid gene expression (2^−ΔCT^ ± SE) of males subjected to knockdown using RNA interference compared with their sperm competition rivals that were injected with elution buffer

Gene (isotig)	RNA_i_	Control	*n*	*p* ^a^
***Tosfp001* (01262)**	0.01 ± 0.00	0.54 ± 0.06	10	<.001
***Tosfp005* (01832)**	0.04 ± 0.01	1.73 ± 0.72	10	.003
***Gagein*** ^b^ **(01709)**	0.02 ± 0.00	2.03 ± 0.17	10	<.001
***Tosfp017* (05129)**	0.06 ± 0.01	8.23 ± 0.97	10	<.001
***Tosfp022* (00444)**	0.05 ± 0.03	4.04 ± 1.03	5	.009
***Tosfp023* (00811)**	0.14 ± 0.02	1.30 ± 0.17	10	<.001
***Tosfp027* (00169)**	0.09 ± 0.21	1.75 ± 0.22	10	<.001

^a^ Probability from nonparametric median test

^b^ *Tosfp011*.

## Discussion

We found that the paternity share of male *T. oceanicus* depended on the expression of *gagein*, a seminal fluid protein found in the ejaculate proteome of this species (ToSfp011, Simmons et al., 2013). *Gagein* expression appeared to contribute to both offensive and defensive roles in sperm competition because the paternity share of the second male to mate increased with the male’s own *gagein* expression relative to the *gagein* expression of his sperm competition rival. RNAi knockdown of *gagein* rendered males mating in the offensive role of second male completely incapable of contributing to the paternity of living offspring. Knockdown of two other seminal fluid protein genes also reduced a male’s paternity share, although natural variation in the expression of these genes had no detectable effect on the paternity share of unmanipulated males.

*Gagein* expression appears to have pleotropic effects on a variety of post-mating phenotypes (Table 2). Knockdown studies have revealed that Gagein supresses a female’s phonotactic behavior (Moschilla et al., 2020), affects the viability of sperm within whole ejaculates (Simmons & Lovegrove, 2017), and the prenatal viability of developing embryos (Simmons & Lovegrove, 2020). Here, we report a clear effect of *gagein* expression on the paternity share of competing males. The pleiotropic effects of sfps on reproductive phenotypes are not unusual. Sex peptide in *Drosophila* has pleiotropic effects on the release of sperm from the female’s sperm storage organs, oviposition, and female sexual receptivity (Wigby et al., 2020; Wolfner, 1997). That knockdown of *ToSfp001*, *ToSfp023,* and possibly *ToSfp022* expression also reduced second male paternity without showing independent effects on the paternity share of unmanipulated males suggests that these three proteins may act indirectly. The functioning of seminal fluid proteins has been found to depend on a network of interacting proteins in *Drosophila*, whereby several different proteins modulate the effect of sex peptide (Findlay et al., 2014; Ram & Wolfner, 2007, 2009). Thus, one possible explanation for the effects we have found in our unmanipulated and knockdown mating trials could be that Gagein is the protein that primarily affects paternity, but ToSfp001, ToSfp023, and possibly also ToSfp022 may be required for Gagein functionality.

**Table 2.** The observed effects of variation in gene expression and/or RNA knockdown of seven *Teleogryllus oceanicus* seminal fluid proteins in males on monogamously or polyandrously mated females

Gene	Sperm viability	Female receptivity	Monogamous (embryo viability)	Polyandrous (relative paternity)	Reference
*ToSfp001*	Positive^a^	Negative	Negative	Positive	1–4
*ToSfp005*	None detected	None detected	None detected	None detected	
*Gagein*	Positive	Negative^a^	Negative	Positive	1–4
*ToSfp017*	Positive^a^	Negative^a^	None detected	None detected	1–4
*ToSfp022*	None detected	Negative	Negative^a^	Positive	1, 2, 4
*ToSfp023*	None detected	Negative^a^	None detected	Positive	1, 4
*ToSfp027*	None detected	None detected		None detected	1,4

^a^Effects intermediate between those genes found to have a significant effect and controls.

1. Simmons & Lovegrove, 2017; 2. Simmons & Lovegrove, 2019; 3. Simmons & Lovegrove, 2020; 4. Moschilla et al., 2020.

There are two sources of variation that can contribute to a male’s share in paternity when assessed from hatched offspring. Males might vary in their ability to fertilize available ova and/or there can be variation among males in the survival of their developing embryos to hatching. Previous studies using noncompetitive, monogamous mating trials have found that knockdown of *gagein* expression in the mates of monogamous females resulted in complete hatching failure (Simmons & Lovegrove, 2020), while increasing expression of *gagein* in unmanipulated males was associated with decreasing pre-hatching embryo viability (Simmons & Lovegrove, 2019). The expression of *ToSfp001* also has negative effects on embryo viability, although to a much lesser extent than *gagein* (Simmons & Lovegrove, 2019). These proteins thereby exhibit the biphasic dose dependence, or hormesis, typical of stimulatory responses found across biological systems (Calabrese, 2013; Calabrese & Mattson, 2017); they are essential for producing offspring but in high doses can be detrimental to their survival. Increased pre-hatching mortality of offspring fathered by *ToSfp001* and *gagein* knockdowns prior to sampling for paternity determination is, therefore, likely to have contributed to the observed reduction in paternity share for these males. The same is not true for *ToSfp023*, however, as knockdown of this protein was found not to affect hatching success in monogamous matings (Simmons & Lovegrove, 2020). Because *gagein* expression is negatively associated with hatching success in monogamously mated females (Simmons & Lovegrove, 2019), all else being equal, we would expect to see negative associations between the paternity share of rival males and their *gagein* expression in polyandrously mated females. In contrast, we found that a male’s paternity share was positively associated with his *gagein* expression relative to that of his sperm competition rival.

The positive effect of *gagein* expression on paternity share in unmanipulated matings suggests that Gagein contributes to paternity share through its effects on competitive fertilization success. If the increased number of fertilizations obtained via the effects of Gagein on competitive fertilization success outweighed the loss of offspring from the detrimental effects of Gagein on offspring development, then males with greater relative *gagein* expression would be expected to have a greater share in the paternity of surviving offspring. Further support for Gagein’s role in competitive fertilization success, and possibly also ToSfp001, ToSfp022, and ToSfp023, comes from the finding that the expression of these proteins increases sperm viability and they are upregulated in response to the risk of sperm competition (Simmons & Lovegrove, 2017). Potential mechanisms underlying the positive effects of these sfps on fertilization success might include effects on sperm transit to and storage in the female sperm storage organ, the longevity of sperm in storage, or their ability to gain access to fertilizable eggs ahead of rival sperm. The effects of Gagein and ToSfp001 on the viability of sperm transferred by males (Simmons & Lovegrove, 2017) could contribute to any or all of these potential mechanisms, and sperm viability is positively associated with competitive fertilization success (García-González & Simmons, 2005). Regardless of the precise mechanism(s) involved, the estimated effect of Gagein on competitive fertilization success of unmanipulated males is undoubtedly conservative given the protein’s negative effect on the survival of fertilized eggs, which will have biased estimates of paternity downwards in favor of rival males with lower *gagein* expression.

Theoretical models of seminal fluid allocation have derived predictions that depend very much on the mode of action of individual seminal fluid proteins in determining male fitness. Our data suggest that Gagein functions to increase competitive fertilization success in both offensive and defensive roles. Seminal fluid games predict that males should increase their expenditure on sfps that increase a male’s competitive fertilization success in offensive and/or defensive roles (Cameron et al., 2007; Dhole & Servedio, 2014), and studies of *Drosophila* offer strong support for these predictions (Hopkins et al., 2019; Wigby et al., 2009). Gene expression studies in which male *T. oceanicus* were exposed to the calling songs of rival males have found that males do increase their expression of several sfps, including *gagein*, *ToSfp001*, *ToSfp023*, and *ToSfp022*, in response to rival song (Simmons & Lovegrove, 2017). Moreover, here, we assayed males for gene expression after they had mated and found that males mating second had higher expression of two sfps, *gagein* and *ToSfp017*, than did their sperm competition rivals who had mated when the female was previously unmated. First and second males had been treated identically prior to mating, suggesting that second males may have increased their sfp gene expressions in response to cues they had detected from the previously mated female. Male *T. oceanicus* have been found to make rapid adjustments in ejaculate quality in response to rival cuticular hydrocarbon residues left on females after mating (Thomas & Simmons, 2008), and *gagein* and *ToSfp017* have been shown to be upregulated in response to rivals (Simmons & Lovegrove, 2017). Since increased gene expression is associated with increased protein production, the increased sfp gene expressions we observed in second males could have reflected a greater allocation of these proteins to females, and subsequent replenishment by the second male.

Our current findings suggest that strategic adjustments in sfp gene expression should enhance a male’s paternity share when females mate multiply, and so increase a male’s relative fitness. However, such adjustments may be detrimental to female fitness given that *gagein* expression has the effect of reducing total offspring production via the negative effect of Gagein on embryo viability (Simmons & Lovegrove, 2019). Just how such conflicting male and female interests affect the evolutionary dynamics of seminal fluid games is an interesting avenue to explore theoretically. For example, we might predict changes in female behavior or physiology that counter the deleterious effects of male sfps on the viability of offspring. In *T. oceanicus*, polyandry has the effect of increasing overall embryo viability to that imparted by the least harmful male (García-González & Simmons, 2007), generating the potential for an interesting feedback mechanism between harm-inducing male sfps that promote competitive fertilization success and the evolutionary maintenance of polyandry. Gene expression studies of *T. oceanicus* have found that males decrease the expression of *gagein* in response to increased sperm competition intensity, defined as the number of males competing for a given set of eggs (Sloan et al., 2018). Multiple mating by females might then be maintained as a mechanism by which they moderate the harmful effects of male sfps on their offspring (Simmons & Lovegrove, 2020). Moreover, we might also expect to find female reproductive tract proteins that counter any negative effects of sfps on female fitness (Wolfner, 2009). In leaf cutter ants, males produce sfps that promote increased motility and death in the sperm of their rivals (Liberti et al., 2018). Sperm mortality, however, is expected to compromise the lifetime fertility of queens because they do not remate after their single nuptial flight. Female reproductive proteins have been identified in leaf cutter ants that cleave the sfps produced by rival males, thereby promoting sperm survival (Dosselli et al., 2019).

Gage (1991) first identified how males strategically adjust the numbers of sperm they transfer to females in response to sperm competition risk. We have identified at least one seminal fluid protein, Gagein, that affects paternity when males compete for fertilizations, the production of which is also adjusted to the current risk and intensity of sperm competition (Simmons & Lovegrove, 2017; Sloan et al., 2018). Ejaculate seminal fluids are rich in proteins, the functions of which are rapidly being uncovered. As we develop our understanding of these functions, we will be able to develop more accurate theoretical models of seminal fluid protein evolution and allocation and the coevolutionary dynamics of sfps with female reproductive physiology.

## Supplementary Material

qrad033_suppl_Supplementary_Material

## Data Availability

The datasets generated and analyzed during this study are available at the Dryad Digital Repository: http://doi:10.5061/dryad.2ngf1vhvc (Simmons & Lovegrove, 2023).

## Footnotes

1 Named in honor of the late Matthew JG Gage who first discovered that males could strategically adjust their ejaculates in response to sperm competition risk (Gage, 1991; Gage & Baker 1991).
