# Supplementary material for: The effect of seminal fluid gene expression on paternity
Source: Evol Lett. 2023 Aug 7;7(5):361–9. doi: 10.1093/evlett/qrad033 (PMC10565890; doi:10.1093/evlett/qrad033)
Supplement: qrad033_suppl_Supplementary_Material [file qrad033_suppl_supplementary_material.pdf]

Supplementary material

The effect of seminal fluid gene expression on paternity

Leigh W Simmons[1,2] and Maxine Lovegrove[1]

Centre for Evolutionary Biology, School of Biological Sciences (M092), The University of Western Australia, Crawley 6009, Australia

[2]E-mail: leigh.simmons@uwa.edu.au

LWS ORCID 0000-0003-0562-1474

KEYWORDS: fertilization success, RNA interference, seminal fluid proteins, sperm competition, *Teleogryllus oceanicus*.

Table S1. Primer sequences and T7* promotor sequence for dsRNA

Gene	Transcript	Forward primer	Reverse primer	Product length
ToSfp001	Isotig01262	TACGAGCCGTTGGAGGACTA	TTTGGAGTGCTTCGTGCGAT	444
ToSfp005	Isotig01832	ACAAGGACGCTGTGGAGATG	ACGAGTGCGAGAAGATGACG	435
Gagein[1]	Isotig01709	AGCCCTCAGACAACAAGCTG	GAAACTCACAGGAGGTCCCG	579
ToSfp017	Isotig05129	CTCAGATACCTCGGCGTCAC	GGCGAAGATCCAGTGGAGAG	372
ToSfp022	Isotig00444	AAGCGAAGAAGGACGAGGTG	CGCTTAAGAGCTGGGCTTCT	524
ToSfp023	Isotig00811	TACCTGAAAGAGCTGGCAGC	TGCTGCAGTCTCAGGTTCAG	465
ToSfp027	Isotig00169	TCGGGGGTGGTTTTTGTCAA	ACGTGAACGAAGATGTTAAACGA	398

* 5'-TAATACGACTCACTATAGGGAGA-3'

[1] *ToSfp011*

Table S2. Primer and reporter sequences for the gene expression assays

Gene	Transcript	Forward primer	Reverse primer	Reporter sequence
Actin	Isotig01761	TGCCATTCTCCGATTGGATCTG	ACGCTCGGTAAGGATCTTCATG	CTGGCCGTGACTTGAC
ToSfp001	Isotig01262	GTAGTGATAGTGCAATCTGGTTACGA	CACAATTTCCCCGCCTTCATC	TTTGACCGCCGT
ToSfp005	Isotig01832	GCGTGTTGCGCCTCTTC	CGTCCTCAGGACTGGAGTCA	CACGCTCACGCACTCC
Gagein[1]	Isotig01709	GCCGAGCAACTGCTGAC	CCGCCTCCTCTTCCTCCTT	TCTCCGCCGCCTCGTC
ToSfp017	Isotig05129	TCGTGGGTGCGGAGTG	CGGCCTTGGCCACGAA	CAGGGCGTGGACCGG
ToSfp022	Isotig00444	GCCGAGCCGGAGGTA	GCTTCTCCACGGCCTTGA	TCCCGCCCCTGCCGC
ToSfp023	Isotig00811	CCAGCAGCTGGTGGAGAAC	GGATGATGTGCCAGTCAACGT	CAACGCCGACCTCATC
ToSfp027	Isotig00169	GGGCGCTACTTGCATCATG	ACGAAATGCCGGCCAGTAA	ACCCCTCCCACGCTCT

[1] *ToSfp011*

Table S3. Analysis of deviance for the full generalised linear model of the effects of relative sfp gene expression on second male paternity.

**Source**	**effect ± SE**	χ^2	***df***	***p***
ToSfp001	1.16 ± 2.23	0.273	1	0.601
ToSfp005	-0.36 ± 0.51	0.499	1	0.480
Gagein[1]	2.90 ± 0.96	12.39	1	<0.001
ToSfp017	-0.02 ± 0.27	0.006	1	0.938
ToSfp022	-0.33 ± 4.07	0.007	1	0.935
ToSfp023	-0.98 ± 1.31	0.569	1	0.450
ToSfp027	0.07 ± 0.61	0.014	1	0.908

Dispersion parameter for quasibinomial family taken to be 11.94
Null deviance: 430.58 on 31 degrees of freedom
Residual deviance: 222.26 on 24 degrees of freedom
McFadden's R^2 = 0.48

[1] *ToSfp011*
